# Malware Identification Method in Industrial Control Systems Based on Opcode2vec and CVAE-GAN

**DOI:** 10.3390/s24175518

**Published:** 2024-08-26

**Authors:** Yuchen Huang, Jingwen Liu, Xuanyi Xiang, Pan Wen, Shiyuan Wen, Yanru Chen, Liangyin Chen, Yuanyuan Zhang

**Affiliations:** 1 The School of Computer Science, Sichuan University, Chengdu 610065, China; yuchen_huang@stu.scu.edu.cn (Y.H.); liujw@stu.scu.edu.cn (J.L.); chenyanru@scu.edu.cn (Y.C.); chenliangyin@scu.edu.cn (L.C.); 2 Pittsburgh Institute, Sichuan University, Chengdu 610065, China; 2021141520064@stu.scu.edu.cn; 3 College of Computer Science and Cybersecurity, Chengdu University of Technology, Chengdu 610059, China; wen.shiyuan@student.zy.cdut.edu.cn

**Keywords:** industrial control system, cybersecurity, malware identification, machine learning

## Abstract

Industrial Control Systems (ICSs) have faced a significant increase in malware threats since their integration with the Internet. However, existing machine learning-based malware identification methods are not specifically optimized for ICS environments, resulting in suboptimal identification performance. In this work, we propose an innovative method explicitly tailored for ICSs to enhance the performance of malware classifiers within these systems. Our method integrates the opcode2vec method based on preprocessed features with a conditional variational autoencoder–generative adversarial network, enabling classifiers based on Convolutional Neural Networks to identify malware more effectively and with some degree of increased stability and robustness. Extensive experiments validate the efficacy of our method, demonstrating the improved performance of malware classifiers in ICSs. Our method achieved an accuracy of 97.30%, precision of 92.34%, recall of 97.44%, and F1-score of 94.82%, which are the highest reported values in the experiment.

## 1. Introduction

With the advancement of the Internet and information technology, the threat of malware to Industrial Control Systems (ICSs) has increased dramatically [1,2]. In ongoing research on malware, identification techniques are continually evolving [3]. Methods based on static analysis, dynamic analysis, and machine learning (hybrid analysis) are emerging [4]. Static analysis is suitable for dealing with unobfuscated malware but has limited effectiveness against techniques like obfuscation and packing [5]. Dynamic analysis performs better with obfuscated code but still suffers from limited applicability and high-performance requirements [6,7]. Given the rapid generation of massive amounts of malware, static and dynamic analysis techniques are gradually becoming inadequate [8,9]. As a result, hybrid analysis methods incorporating machine learning have emerged [10,11,12]. These methods significantly enhance the performance of large-scale malware identification by extracting features from large datasets and leveraging machine learning for classification and clustering. However, despite the theoretical applicability of these machine learning-based techniques to ICS, their effectiveness in practical applications is limited due to the smaller datasets available for ICS [3,13]. They need further optimization to meet the specific needs of ICS.

Effective malware classification and identification using machine learning necessitate a large number of training samples to achieve optimal results [14,15]. However, current research primarily focuses on platforms such as Windows, iOS, and Android [16,17], leaving ICSs with relatively fewer samples [3]. If existing methods are directly applied to ICSs, they will likely fail to achieve satisfactory accuracy due to insufficient training samples [13,18]. Additionally, most existing research focuses on improving the identification performance of classifiers but falls short in enhancing their robustness against attacks [19]. Malware authors can generate attack samples through subtle code modifications, such as indirect addressing, inserting useless code, and reordering, leading to classifier misjudgments [20,21]. These attacks pose significant security risks to malware classifiers in ICSs. In summary, existing machine learning models face two primary challenges in recognizing malware in ICS. First, the limited number of training samples results in poor model accuracy. Second, the lack of diversity in training samples leads to models’ lack of stability and robustness.

To address the challenges mentioned above, we propose an innovative method for Data-Enhanced Malware Identification (DEMI) specifically designed for ICSs. This method combines the opcode2vec method based on preprocessed features with a Conditional Variational AutoEncoder–Generative Adversarial Network (CVAE-GAN) [22,23] to generate malware samples. Then, the method utilizes the original and generated malware to train the classifier. During the generation of malware samples, our method utilizes a unique preprocessing method and opcode2vec for effective feature extraction, combined with the latent space learning capability of Conditional Variational AutoEncoder (CVAE) [24] and the high-quality generative ability of Generative Adversarial Network (GAN) [25]. The method generates a large-scale, diverse set of malware samples tailored specifically for Industrial Control Systems. The generated diverse samples significantly enrich the training dataset, compensating for the limitations of existing samples in terms of features and categories. During the identification of malware samples, these samples are used to train the classifier. After training, the classifier not only improves its performance in identifying various types of malware in ICSs but also enhances its robustness against metamorphic attacks.

The main contributions of our work are summarized as follows:

- To address the challenge of the smaller quantity and limited diversity of malware samples in ICSs, our work proposes a novel malware generation method that combines the opcode2vec method based on preprocessed features and CVAE-GAN After the unique preprocessing, opcode2vec converts each opcode into a word vector. The preprocessing ensures that the extracted features remain both simple and effective. Simultaneously, CVAE-GAN leverages the latent space learning capabilities of CVAE with the high-quality generation capabilities of GAN to produce a large and diverse set of malware samples that meet specified conditions. These enhanced samples significantly enrich the training dataset, compensating for the deficiencies of existing samples in terms of features and categories.
- To address the challenges of low accuracy, instability, and lack of robustness in existing classifier models, our work has developed a malware classifier based on an enhanced dataset. We conducted comprehensive analysis and experiments, focusing on the model trained using the DEMI. The experimental results demonstrate that the model, based on the DEMI proposed in our work, shows a significant advantage in accuracy, particularly in scenarios with a limited number of samples.

The remainder of this paper is structured as follows. Section 2 reviews the related work. Section 3 elaborates on the proposed method. Section 4 details the experiments and analysis. Section 5 discusses the limitations and potential future work. Section 6 concludes our work.

## 2. Related Work

In this section, we discuss related work on ICS security, focusing on malware and identification methods.

### 2.1. Malware

With the rapid development of the Internet, the integration of information technology and industrialization has significantly intensified. To enable interconnectivity between various systems, ICSs increasingly employ information and communication network technologies, which pose severe challenges to industrial control system security. Stuxnet caused devastating damage to Iran’s uranium enrichment facilities [26]. The BlackEnergy and Crashoverride malware attacked the Ukrainian power grid in 2015 [27] and 2016 [28], respectively. BlackEnergy simultaneously attacked three substations, resulting in power outages for over 200,000 users for several hours [27]. Crashoverride also shut down a substation supplying power to parts of Kyiv for about an hour [28]. Other notable malware include Havex, designed to infiltrate industrial networks [29], and Trisis, specifically targeting Triconex safety systems [30]. The examples mentioned above, including Stuxnet, BlackEnergy, Crashoverride, Havex, and Trisis, are quintessential examples of malware in ICSs.

### 2.2. Identification Methods

In the realm of malware identification, current research trends increasingly incorporate machine learning. Chu et al. proposed an industrial control intrusion detection method based on a deep learning model that leverages the communication processes of the Modbus protocol combined with a GoogLeNet–long short-term memory model to achieve efficient intrusion detection and classification [31]. Krithivasan et al. introduced a novel anomaly detection technique, EPCA-HG-CNN, which utilizes Enhanced Principal Component Analysis and Convolutional Neural Networks based on HyperGraphs to identify abnormal behaviors in ICS [32]. Selim et al. applied various machine learning methods to classify anomalous events in critical water infrastructure within the Industrial Internet of Things (IIoT) framework [33]. Hassini et al. simulated power generation facilities using a hardware-in-the-loop testbed and implemented various attacks with the dataset. Their findings revealed that AdaBoost exhibited the best accuracy and performance among the machine learning algorithms, while Convolutional Neural Networks (CNNs) outperformed other deep learning algorithms in accuracy [34]. Handa et al. developed a scalable fair clustering algorithm to establish a Fairlet Decomposition (FD) model, which they validated using three different datasets: Internet of Things (IoT), Secure Water Treatment (SWaT), and ICSs. The experimental results indicated that the FD model performed best on the IoT dataset [35]. A comprehensive survey of existing methods indicates that most machine learning-based ICSs’ malware identification works focus on optimizing algorithms based on existing datasets. However, as highlighted in the literature [3], challenges such as the scarcity of high-quality realistic datasets and the prevalence of adversarial attacks continue to impede ICS security. The datasets used for training, testing, and evaluating machine learning methods in ICSs are often outdated and unrealistic, reflecting only specific types of attacks. Additionally, the risk of exposing sensitive information deters the sharing of real system datasets. These issues can potentially be addressed by leveraging the powerful data generation capabilities of CVAE-GAN.

## 3. Method

In this section, we provide a comprehensive introduction to DEMI, a method designed to tackle the challenges of insufficient sample size and lack of diversity in the training process, which can result in models with low accuracy and lack of stability and robustness. DEMI comprises three primary components. Firstly, we perform feature extraction from malware samples using the opcode2vec method based on preprocessing. Secondly, we enhance the industrial control system (ICS) malware dataset by generating new malware samples through CVAE-GAN. Lastly, we identify malware samples by training a novel ICS malware identification model using the augmented dataset. This section first provides an overview of the DEMI framework in Section 3.1, then delves into each component in Section 3.2, Section 3.3 and Section 3.4, and concludes with a discussion of the evaluation metrics in Section 3.5.

### 3.1. The Overall Structure of DEMI

**Figure 1:**
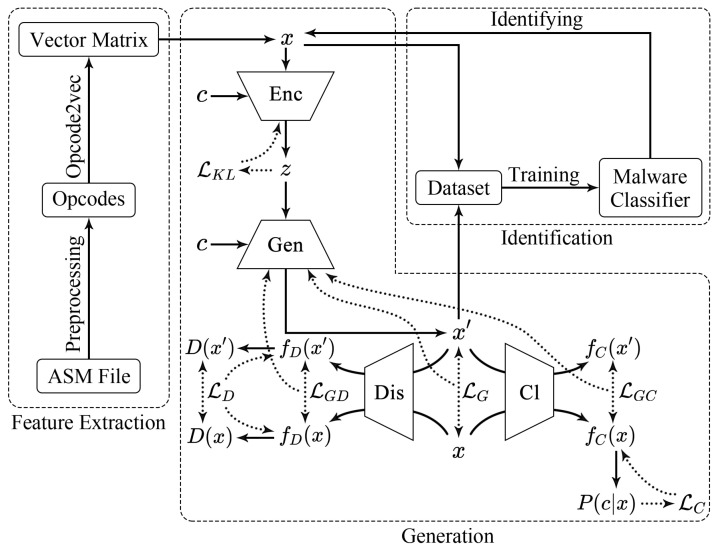
The overall structure of DEMI.

Overall, DEMI consists of three main components to deal with malware: feature extraction, generation, and identification. During the feature extraction process, preprocessing is primarily focused on opcodes. Initially, opcodes are extracted from ASM files. Subsequently, key opcodes are selected based on information entropy. Finally, these opcodes are converted into vector matrices using opcode2vec, which serve as samples for the subsequent generation and identification stages. In the generation phase, the work is based on the CVAE-GAN model. First, the CVAE-GAN model is trained to enhance the quality of the generated samples. Then, the trained model is used to generate high-quality new samples. In the identification phase, there are two main tasks: training a CNN-based classifier and identifying malware samples. Initially, the CNN classifier is trained, and then the trained CNN-based classifier is used for malware sample identification. Our method, DEMI, is illustrated in the accompanying Figure 1.

### 3.2. The Feature Extraction of DEMI

This subsection introduces the opcode2vec method based on preprocessed features. It is divided into three parts: extraction of opcodes from ASM files, selection of opcode based on information entropy, and opcode word vector computation using opcode2vec. Each of these components is elaborated upon in detail.

#### 3.2.1. The Extraction of Opcodes

In malware analysis, disassembly tools are typically used to generate ASM files that provide the assembly code of the malware. A typical disassembled ASM file structure generally includes the following sections: the header, the text segment (.text), and the data segment (.data). The header mainly contains comments such as the filename, generation time, and compiler information. However, depending on the disassembly tool and selected options, the presence and content of the header may vary or even be absent. The data segment contains the program’s static data, such as initialized variables and constants, which are generally of limited significance for analyzing code behavior. Unlike them, the text segment is the core of the disassembled file and contains the executable instructions of the code. Therefore, our focus should be on extracting and analyzing the text segment.

For the text segment, it is necessary to extract individual functions from the assembly file sample based on the function identifiers in the ASM file. Specifically, the process involves the following steps: First, identify the boundaries of each function, including the start and end. The start of a function is typically marked by the function name and address, while the end is usually indicated by a return instruction. Second, read each line of the ASM file, recognize the function names and addresses, and record the starting and ending lines of each function. Finally, extract the code segment for each function based on the recorded line numbers.

Additionally, for each function within a text segment, it is necessary to remove the function parameters and extract only the opcodes. This step is crucial for enhancing the robustness of malware identification. Research has shown that even with the same compilation, the same function can exhibit significant differences in register and jump locations due to variations in its position within the malware source code or differences in parameter naming. As a result, malware authors frequently create new variants by modifying source code, adjusting function positions, or changing parameters, which are techniques characteristic of metamorphic attacks. Consequently, our work focuses on retaining only the opcodes.

#### 3.2.2. The Selection of Opcodes

A subset of opcodes with high information entropy can provide better accuracy and performance than a larger set of opcodes [36]. To improve model efficiency and reduce the dimensionality of feature representations, our work selected opcodes with information entropy higher than a threshold *e* for feature extraction. The formula for the threshold *e* is defined as (1).

(1) $$e\left(op_{k}\right)=\frac{\sum_{i=1}^{z}\left(\frac{\sum_{x_{j}\in f_{i}}p\left(op_{k}|x_{j}\right)}{n_{j}}/\frac{\sum_{x_{j}\notin f_{i}}p\left(op_{k}|x_{j}\right)}{N-n_{j}}\right)}{z}$$

Let $OP=(op_{1},op_{2},\ldots ,op_{y})$ represent the set of all opcodes, where *y* denotes the total number of opcodes. Let $F=(f_{1},f_{2},\ldots ,f_{z})$ represent the set of malware families, where *z* denotes the total number of malware families. Let $x_{j}$ denote a malware instance. Let $p(op_{i}|x_{j})$ be the probability function of $op_{i}$ in $x_{j}$. $n_{j}$ represents the number of instances of $f_{i}$ malware family, and *N* represents the number of the total instances. The threshold *e* should be determined by considering the feature dimension and the accuracy of different malware. In our work, we found that when the threshold e=0.2, the comprehensive performance was optimal, resulting in 266 selected opcodes.

#### 3.2.3. The Word Vector Computation of Opcodes

**Figure 2:**
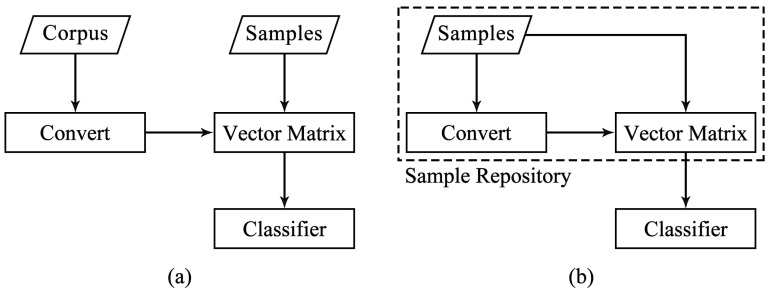
The comparison of corpus between word2vec and opcode2vec: (**a**) Word2vec. (**b**) Opcode2vec.

In our work, there is no additional related corpus available, making the conventional word2vec method infeasible. Therefore, we propose the opcode2vec method based on preprocessed features. The fundamental difference between opcode2vec and word2vec is illustrated in Figure 2. This method converts opcodes into corresponding word vectors, where the word vectors represent the semantic relationships between opcodes. Opcode2vec, based on preprocessed features, can improve the accuracy and effectiveness of malware feature representation. Essentially, opcode2vec is trained on the corpus of each sample to obtain the word vectors for each opcode in the sample, which can be represented as (2).

(2) $$V_{s}^{i}=\left(x_{1}^{i},x_{2}^{i},\cdots ,x_{n}^{i}\right)$$

Here, $V_{s}^{i}$ denotes the word vector of the *i*-th opcode in the malware sample *s*, $x_{j}^{i}$ represents the value of the *j*-th dimension of the word vector, and *n* is the dimension of the word vector. In our work, we set the dimension of the word vectors equal to the number of opcodes, i.e., n=266. Finally, we arrange the opcodes in descending order based on information entropy to form the feature vector matrix. Consequently, the opcode-based malware feature extraction is completed, resulting in a 266×266 feature matrix.

### 3.3. The Generation of DEMI

**Figure 3:**
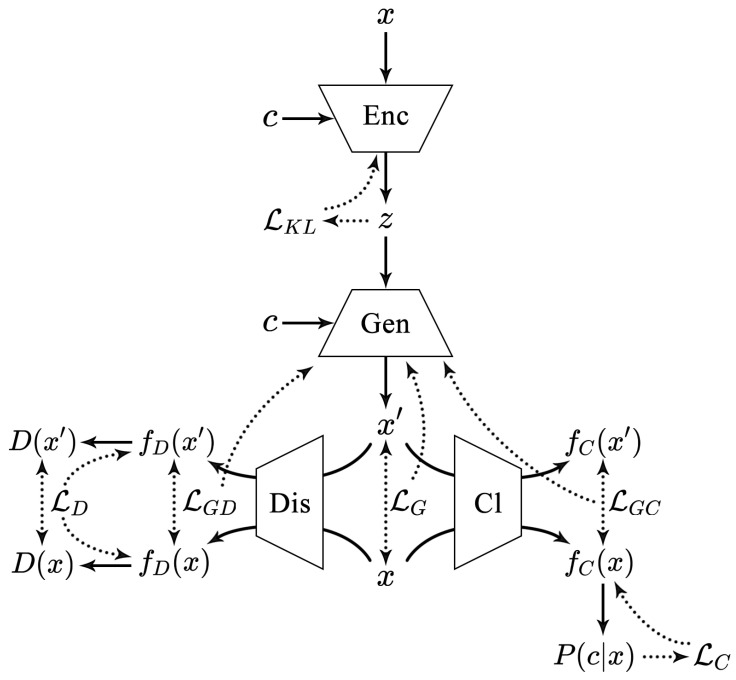
The overall structure of CVAE-GAN [22].

Based on feature extraction, our work utilizes CVAE-GAN to generate large-scale and diverse samples. The CVAE-GAN structure is illustrated in Figure 3.

#### 3.3.1. Component Definitions

1. Encoder (Enc)
  - Function: Encodes the input malware sample *x* into the latent variable *z*.
  - Input: *x* (input sample), *c* (category).
  - Output: *z* (latent variable).
  - Mathematical Operation: z=Enc(x,c)
  - Associated Loss: $L_{KL}$ (Kullback-Leibler ( KL) divergence loss).
2. Generator (Gen)
  - Function: Acts both as the decoder in the VAE and the generator in the GAN, decoding the latent variable *z* into the generated sample x’, thus generating a malware sample belonging to category *c*.
  - Input: *z* (latent variable), *c* (category).
  - Output: x’ (generated sample).
  - Mathematical Operation: $x^{\prime }=Gen\left(z,c\right)$
  - Associated Loss: $L_{G}$ (loss function for the generator part, including $L_{GD}$ and $L_{GC}$).
3. Discriminator (Dis)
  - Function: Determines whether the input sample is real or fake.
  - Input: *x* (real sample), x’ (generated sample).
  - Output: D(x), D(x’) (discrimination results).
  - Mathematical Operation: D(x)=Dis(x), $D\left(x^{\prime }\right)=Dis\left(x^{\prime }\right)$
  - Associated Loss: $L_{D}$ (loss function for the discriminator part).
4. Classifier (Cl)
  - Function: Measures the posterior probability P(c|x). The classifier in CVAE-GAN is used to optimize the parameters of the generator through the losses $L_{C}$ and $L_{GC}$.
  - Input: *x* (real sample), $x^{\prime }$ (generated sample).
  - Output: P(c|x), $P(c|x^{\prime })$ (classification results).
  - Mathematical Operation: P(c|x)=Cl(x), $P\left(c|x^{\prime }\right)=Cl\left(x^{\prime }\right)$
  - Associated Loss: $L_{C}$ (loss function for the classifier part).
5. Category (c)
  - Function: The given category corresponding to the malware sample *x*, indicating which malware family it belongs to.

#### 3.3.2. Loss Functions

-
$L_{KL}$: KL divergence loss for the VAE network, representing the difference between the latent vector distribution P(z) and the predefined distribution.
-
$L_{G}$: Loss function for the generator part of the GAN, which includes both the loss functions $L_{GD}$ and $L_{GC}$.
-
$L_{D}$: Loss function for the discriminator part of the GAN.
-
$L_{C}$: Loss function for the classifier part.
-
$L_{GD}$ and $L_{GC}$: Both are components of the loss function for the generator part of the GAN.

#### 3.3.3. Training Phase

During the training phase, the model learns to encode the input sample *x* into a latent representation *z*, generate a new sample $x^{\prime }$ from *z*, and discriminate between real and generated samples. The role of the classifier is to guide the generator to produce $x^{\prime }$ corresponding to the label *c* through the loss functions.

1. Encoder:
z=Enc(x,c)
$$L_{KL}=KL\left(q\left(z|x,c\right)\|P_{z}\right)$$
2. Generator:
$$x^{\prime }=Gen\left(z,c\right)$$
$$L_{G}=\frac{1}{2}\left(∥x-x^{\prime }{∥}_{2}^{2}+L_{GD}+L_{GC}\right)$$
3. Discriminator:
D(x)=Dis(x)
$$D\left(x^{\prime }\right)=Dis\left(x^{\prime }\right)$$
$$L_{D}=-\left(\log D\left(x\right)+\log\left(1-D\left(x^{\prime }\right)\right)\right)$$
4. Classifier:
P(c|x)=Cl(x)
$$P\left(c|x^{\prime }\right)=Cl\left(x^{\prime }\right)$$
$$L_{C}=-\log P\left(c|x\right)$$

In generator training, the loss function between the input sample and the generated sample must incorporate the losses from both the discriminator and the classifier. In addition to the original loss function, we introduced paired feature matching losses as (3).

(3) $$L_{G}=\frac{1}{2}\left(∥x-x^{\prime }{∥}_{2}^{2}+L_{GD}+L_{GC}\right)$$

where $L_{GD}$ and $L_{GC}$ are feature matching losses, detailed in (4) and (5). Here, $f_{D}$ and $f_{C}$ represent the features from the intermediate layers of the discriminator and the classifier, respectively.

(4) $$L_{GD}=\frac{1}{2}{∥\frac{1}{m}\sum_{i=1}^{m}f_{D}\left(x\right)-\frac{1}{m}\sum_{i=1}^{m}f_{D}\left(x^{\prime }\right)∥}_{2}^{2}$$

(5) $$L_{GC}=\frac{1}{2}\sum_{c}{∥f_{C}^{c}\left(x\right)-f_{C}^{c}\left(x^{\prime }\right)∥}_{2}^{2}$$

Feature matching loss stabilizes the training process by extracting features from real and fake samples across different scales. The final loss function for the adversarial generation of malware samples is given in (6).

(6) $$L_{G}=\frac{1}{2}\left(∥x-x^{\prime }{∥}_{2}^{2}+∥f_{D}\left(x\right)-f_{D}\left(x^{\prime }\right){∥}_{2}^{2}+{∥f_{C}\left(x\right)-f_{C}\left(x^{\prime }\right)∥}_{2}^{2}\right)$$

In summary, the total loss function for the adversarial generation of malware samples during the training phase is presented in (7).

(7) $$L=L_{D}+L_{C}+\lambda_{1}L_{KL}+\lambda_{2}L_{G}+\lambda_{3}L_{GD}+\lambda_{4}L_{GC}$$

The overall training objective is to minimize this loss function, where the parameters $\lambda_{1}$, $\lambda_{2}$, $\lambda_{3}$, and $\lambda_{4}$ are weight coefficients used to adjust the relative importance of different loss terms in the total loss function. Each parameter represents the influence of a specific loss term.

During the training process, parameters θ are continuously updated using gradient descent based on the results of the loss function. The update sequence is as follows:

(8) $$\begin{array}{cc} \theta_{C} & \leftarrow \theta_{C}-\alpha_{C}\nabla_{\theta_{C}}\left(L_{C}\right) \end{array}$$

(9) $$\begin{array}{cc} \theta_{D} & \leftarrow \theta_{D}-\alpha_{D}\nabla_{\theta_{D}}\left(L_{D}\right) \end{array}$$

(10) $$\;\;\;\;\;\;\;\;\begin{array}{cc} \theta_{G} & \leftarrow \theta_{G}-\alpha_{G}\nabla_{\theta_{G}}\left(\lambda_{2}L_{G}+\lambda_{3}L_{GD}+\lambda_{4}L_{GC}\right) \end{array}$$

(11) $$\;\;\;\;\begin{array}{cc} \theta_{E} & \leftarrow \theta_{E}-\alpha_{E}\nabla_{\theta_{E}}\left(\lambda_{1}L_{KL}+\lambda_{2}L_{G}\right) \end{array}$$

Here, $\theta_{C}$ denotes the parameters of the classifier; $\theta_{D}$ denotes the parameters of the discriminator; $\theta_{G}$ denotes the parameters of the generator; and $\theta_{E}$ denotes the parameters of the encoder. α is the learning rate in gradient descent.

This process is repeated until $\theta_{G}$ converges, indicating that the generator has reached its optimal state, thereby completing the model training phase for the adversarial generation of malware samples. The training algorithm for this phase is presented in Algorithm 1.
**Algorithm 1** Training Pipeline of Generation.**Require:** Number of samples: *m*. Initialization parameters of encoder (*E*): $\theta_{E}$. Initialization parameters of generator (*G*): $\theta_{G}$. Initialization parameters of discriminator (*D*): $\theta_{D}$. Initialization parameters of classifier (*C*): $\theta_{C}$. Learning rates: $\alpha_{C}$, $\alpha_{D}$, $\alpha_{G}$, $\alpha_{E}$. Weighting factors: $\lambda_{1}$, $\lambda_{2}$, $\lambda_{3}$, $\lambda_{4}$. 1: **while** $\theta_{G}$ has not converged **do** 2:     Samples $\left(x_{r},c_{r}\right)$ from the real data distribution $P_{r}$ 3: $L_{C}\leftarrow -\log P\left(c_{r}|x_{r}\right)$ 4: $z\leftarrow E(x_{r},c_{r})$ 5:     Samples $z_{p}$ and $c_{p}$ from the predefined distribution $P_{z}$ 6: $L_{KL}\leftarrow KL\left(q\left(z|x_{r},c_{r}\right)\|P_{z}\right)$ 7: $x_{f}\leftarrow G\left(z,c_{r}\right)$ 8: $x_{p}\leftarrow G\left(z_{p},c_{p}\right)$ 9: $L_{D}\leftarrow -\left(\log D\left(x_{r}\right)+\log\left(1-D\left(x_{f}\right)\right)+\log\left(1-D\left(x_{p}\right)\right)\right)$10:     Compute the feature center of $x_{r}$ and $x_{p}$11: $L_{GD}\leftarrow \frac{1}{2}{∥\frac{1}{m}\sum_{i=1}^{m}f_{D}\left(x_{r}\right)-\frac{1}{m}\sum_{i=1}^{m}f_{D}\left(x_{p}\right)∥}_{2}^{2}$12:     Compute the feature center of $x_{r}$ and $x_{p}$ with respect to $c_{i}$ separately13: $L_{GC}\leftarrow \frac{1}{2}\sum_{c_{i}}{∥f_{C}^{c_{i}}\left(x_{r}\right)-f_{C}^{c_{i}}\left(x_{p}\right)∥}_{2}^{2}$14: $L_{G}\leftarrow \frac{1}{2}\left(∥x_{r}-x_{f}{∥}_{2}^{2}+∥f_{D}\left(x_{r}\right)-f_{D}\left(x_{f}\right){∥}_{2}^{2}+{∥f_{C}\left(x_{r}\right)-f_{C}\left(x_{f}\right)∥}_{2}^{2}\right)$15: $\theta_{C}\leftarrow \theta_{C}-\alpha_{C}\nabla_{\theta_{C}}\left(L_{C}\right)$16: $\theta_{D}\leftarrow \theta_{D}-\alpha_{D}\nabla_{\theta_{D}}\left(L_{D}\right)$17: $\theta_{G}\leftarrow \theta_{G}-\alpha_{G}\nabla_{\theta_{G}}\left(\lambda_{2}L_{G}+\lambda_{3}L_{GD}+\lambda_{4}L_{GC}\right)$18: $\theta_{E}\leftarrow \theta_{E}-\alpha_{E}\nabla_{\theta_{E}}\left(\lambda_{1}L_{KL}+\lambda_{2}L_{G}\right)$19: **end while**

#### 3.3.4. Inference Phase

During the inference phase, CVAE-GAN uses the parameters learned during the training phase to generate new samples $x^{\prime }$, mainly involving the encoder and generator.

1. Encoder:
z=Enc(x,c)
2. Generator:
$$x^{\prime }=Gen\left(z,c\right)$$
3. Discriminator and Classifier: The discriminator and classifier do not directly participate in the inference phase. However, during the training phase, they optimize the generator’s parameters to ensure the quality and consistency of the generated samples.

### 3.4. The Identification of DEMI

In the previous subsection, we trained a model capable of generating high-quality adversarial samples of malware. This model allows the generation of a large and diverse set of new samples using only a small amount of original malware samples. These generated samples, together with the original samples, are used to train the malware classifier based on CNN proposed in this subsection, whose parameters are shown in Table 1. Specifically, we split the original dataset into 80% for training and 20% for testing. All generated samples are added to the training set to train the classifier. As described in Section 3.2.3, each processed malware sample is represented by a 266×266 feature matrix.

### 3.5. The Evaluation of DEMI

To assess the classifier in DEMI, we used several evaluation metrics, including accuracy, precision, recall, and F1-score. Below are the definitions and calculation methods for each metric, where TP is true positive, TN is true negative, FP is false positive, and FN is false negative.

1. Accuracy: Accuracy is the ratio of correctly predicted instances (both true positives and true negatives) to the total number of instances.
$$\mathrm{Accuracy}=\frac{TP+TN}{TP+TN+FP+FN}$$
2. Precision: Precision is the ratio of correctly predicted positive instances to the total predicted positives.
$$\mathrm{Precision}=\frac{TP}{TP+FP}$$
3. Recall: Recall, also known as sensitivity, is the ratio of correctly predicted positive instances to all instances that are actually positive.
$$\mathrm{Recall}=\frac{TP}{TP+FN}$$
4. F1-score: The F1-score is the harmonic mean of precision and recall, providing a balanced metric that considers both false positives and false negatives.
$$F1\text{-}\mathrm{score}=2\times \frac{\mathrm{Precision}\times \mathrm{Recall}}{\mathrm{Precision}+\mathrm{Recall}}$$

## 4. Experiment and Analysis

In this section, we present the experiments conducted in our work, including details about the dataset, experimental setup, and results. For the results, we provide the outcomes of the experiments performed solely on DEMI and compare DEMI’s performance with other methods.

### 4.1. Dataset and Experimental Setup

**Figure 4:**
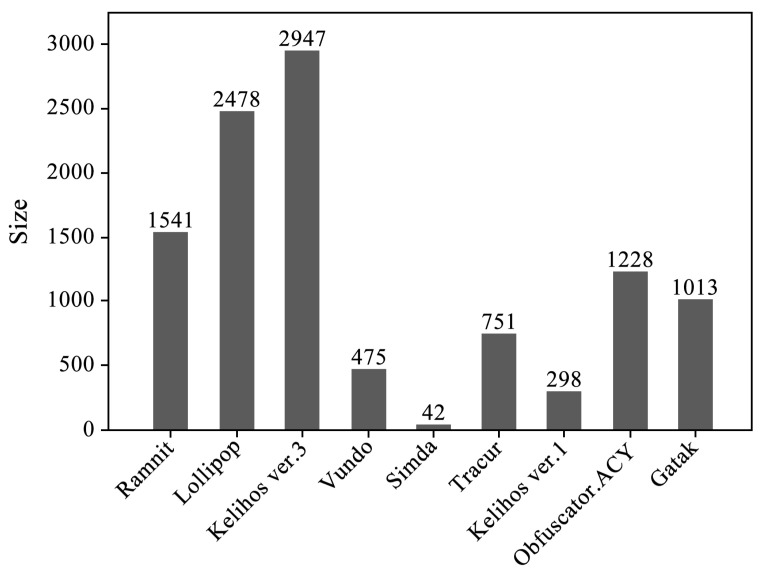
Distribution of malware family sample sizes.

Because our dataset is not readily available to the public, we used the dataset from the Microsoft Malware Classification Challenge [37] for preliminary validation of the proposed model’s validity. This allows us to demonstrate the effectiveness of our model while we work on obtaining and integrating a dataset specifically tailored to ICS malware. The dataset contains 10,868 labeled samples (10,773 of which are actually usable), each belonging to 1 of 9 different malware families. Each sample consists of two types of files: (1) an asm file generated using the IDA disassembler tool, containing logs of various metadata extracted from the binary file, such as function calls and strings; (2) a byte file representing the hexadecimal representation of the file’s binary content (excluding the PE header). The 10,868 sample dataset used in our work is fully labeled. Each sample contains a unique identifier (ID), a 20-character hash value that uniquely identifies the sample. This ID corresponds to a Class value, an integer from 1 to 9, representing the malware family to which the sample belongs. The distribution of the number of samples in each malware family across the entire dataset is shown in Figure 4. The experimental setup is as follows: An Intel(R) Core(TM) i7-4790K CPU operating at 4.00 GHz was used as the processor. The system is equipped with 8.00 GB of memory and runs a 64-bit operating system on an x64-based processor. The experiments were conducted on a Windows 10 operating system. The Python version used for the experiments is 3.7.7, along with Numpy version 1.13.1 and Sklearn version 1.0.2. PyCharm served as the integrated development environment (IDE) for the experimental work.

### 4.2. Results of DEMI Experiment

This subsection presents the experimental results of DEMI in two parts. The first part covers the full-sample experiment, evaluating whether the newly generated samples can enhance the classifier’s identification performance. The second part focuses on the few-sample experiment, assessing whether the newly generated samples can consistently stabilize the classifier’s identification performance under varying original sample sizes.

#### 4.2.1. Results of Full-Sample Training

We propose a model that enhances the dataset by generating samples based on the original samples. A total of five rounds of experiments were conducted, where the number of real samples for each family was kept constant in each round, corresponding to all the original samples in the dataset. In each round, the number of generated samples was varied, with the number of generated samples in each round being 120% (rounded to the nearest integer) of the number generated in the previous round. In the first round, the number of generated samples was 20% of the number of original samples. The number of real and generated samples for each family in each round of the experiment is shown in Table 2.

**Figure 5:**
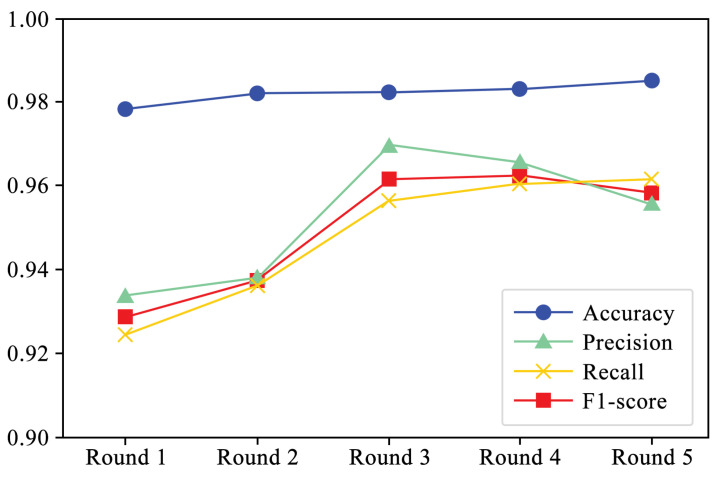
Performance trends per experiment round.

**Figure 6:**
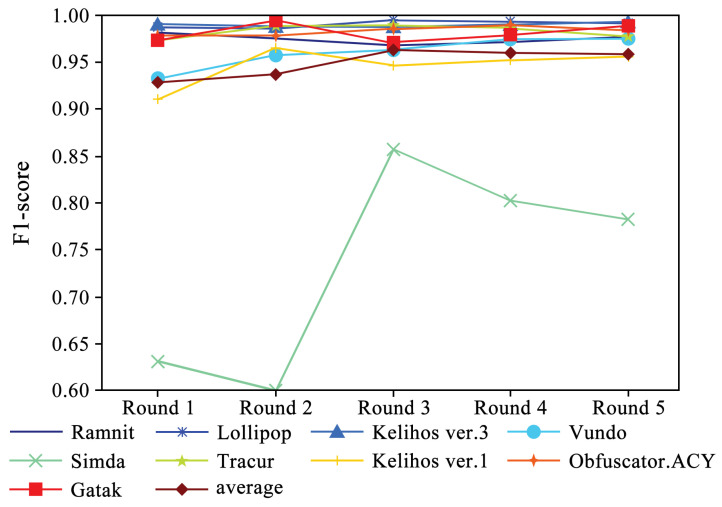
F1-score trends of different malware families.

**Figure 7:**
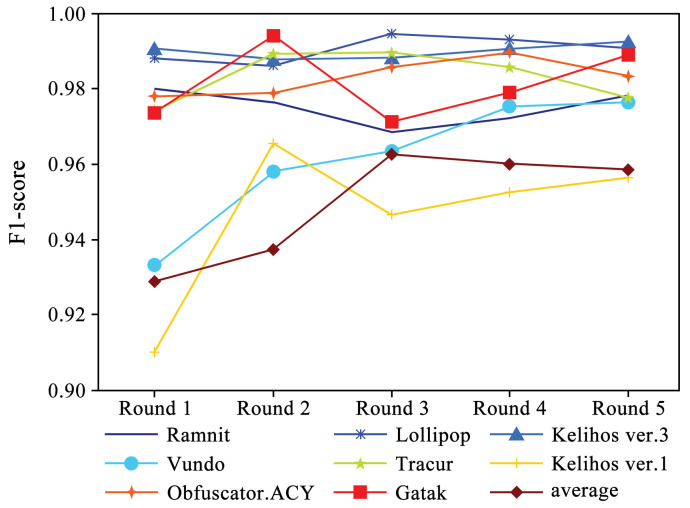
F1-score trends of different malware families (excluding Simda).

Figure 5 shows the performance of four evaluation metrics in each round of experiments. It can be observed that accuracy and recall exhibit a slight upward trend. Additionally, Figure 6 and Figure 7 present the F1-scores of different families. As shown in Figure 6, the F1-score of each family displays a slight upward trend. The Simda family shows significant fluctuations, but overall, it also exhibits an upward trend. To better understand the performance trends of each family, Figure 7 enlarges the y-axis after excluding the Simda family. It can be seen that families such as Gatak, Tracur, and Lollipop, which originally had higher F1-scores, fluctuate within a small range. In contrast, families such as Vundo and Kelihos ver.1, which initially had lower F1-scores, show an upward trend in identification performance as the number of generated samples increases in each round. Based on the experimental results, it is evident that DEMI can effectively improve identification performance by increasing the number of generated adversarial samples.

#### 4.2.2. Results of Few-Sample Training

On the basis of the full-sample, we incrementally reduce the number of samples for each malware family and train the model based on DEMI under few-sample conditions to obtain experimental results. The results demonstrate the effectiveness of the proposed method with limited samples. The experiment consists of eight rounds, with the sample size for each round comprising nine malware families. Each family’s sample size is 80% (rounded to the nearest integer) of the sample size from the previous round. Among them, 80% are used for model training, and the remaining 20% are used for testing. Considering that the Simda family has only 42 samples in total, and its highest identification accuracy in the experiment was only 80%, the further reduction would severely affect the overall identification accuracy. Therefore, the number of samples for this malware family is kept constant at 42 samples in each round. In each round of the experiment, DEMI generates adversarial samples for each malware family. The number of generated samples is 20% of the current number (rounded to the nearest integer). Additionally, only adversarial samples for the Simda family were generated in the first round. The number of samples in each round is shown in Table 3.

**Figure 8:**
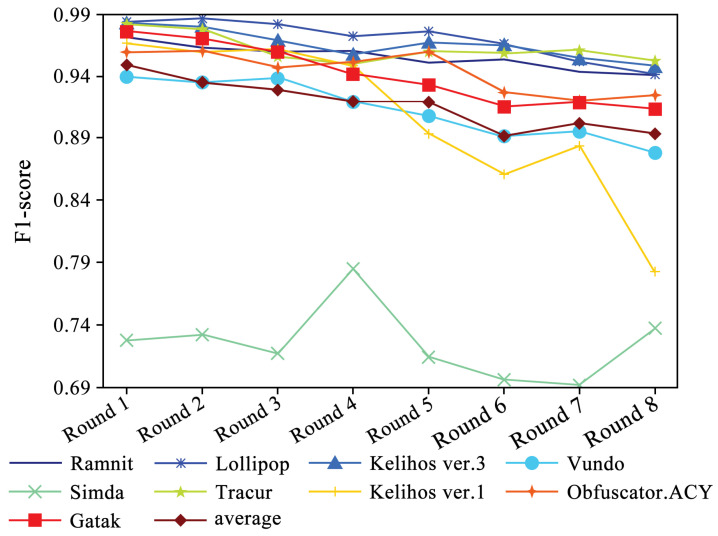
F1-score trends across eight rounds of few-sample experiments.

The identification accuracy of each family in each round based on the DEMI classifier is shown in Figure 8. Although the identification accuracy of each family decreases as the number of samples decreases, the trend is relatively smooth. Despite the number of Simda family samples remaining unchanged, its accuracy fluctuates significantly due to the reduction in samples from other families, showing an overall downward trend. The experimental results indicate that thanks to the data augmentation provided by DEMI, the classifier’s performance remains relatively stable even as the number of samples continues to decrease.

### 4.3. Comparison of Different Methods

Similarly, this subsection is divided into two parts to compare different methods. The first part compares the performance of different methods based on full-sample experiments. The second part evaluates the stability of these methods through comparisons in few-sample experiments.

#### 4.3.1. Comparison of Results for Full-Sample Training

**Figure 9:**
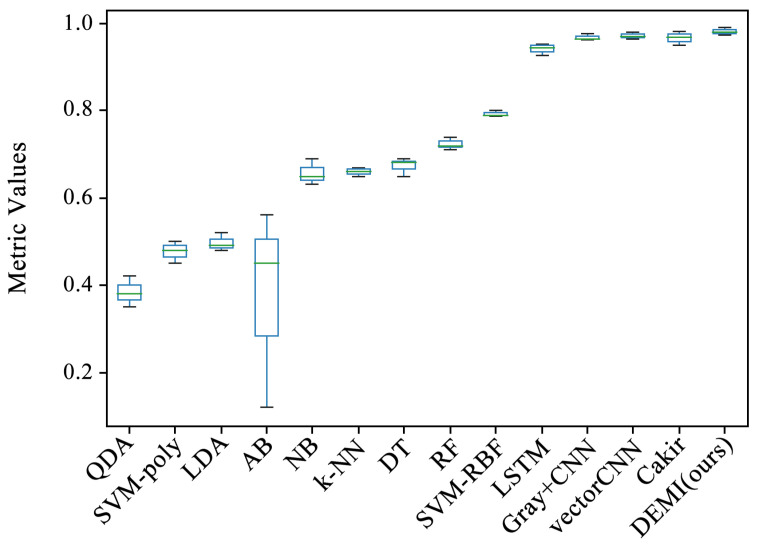
Comparison of all methods with proposed DEMI.

**Figure 10:**
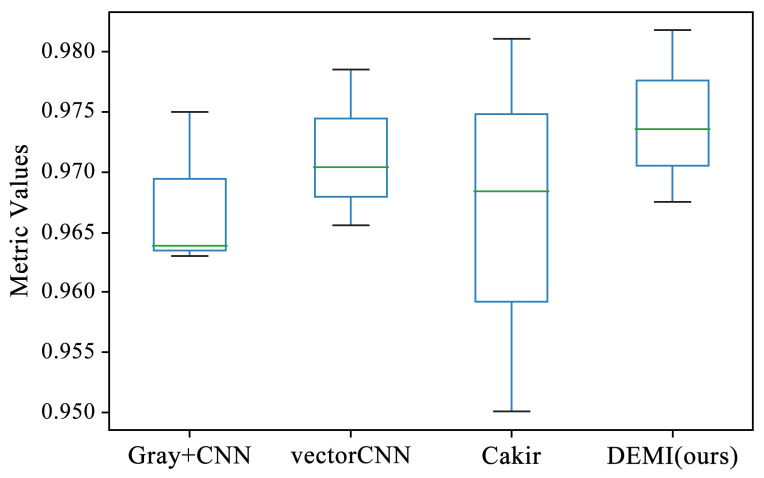
Comparison of SOTA methods with proposed DEMI.

All malware samples were used for model training, and a ten-fold cross-validation experiment was conducted to obtain the experimental results of each model under full-sample conditions. This subsection compares several traditional machine learning algorithms, such as Quadratic Discriminant Analysis (QDA), Support Vector Machine (SVM) with polynomial kernel, Linear Discriminant Analysis (LDA), AdaBoost, Naive Bayes (NB), k-Nearest Neighbors (k-NN), Decision Tree (DT), Random Forest (RF), and SVM with Radial Basis Function (RBF) kernel, all of which are available in the Sklearn library. Additionally, we compared methods such as Gray+CNN (directly converting code to grayscale images), vectorCNN (using word2vec for feature extraction), and Cakir’s method [38]. After performing ten-fold cross-validation on these models, the accuracy box-plots were obtained as shown in Figure 9. It can be seen that the accuracy of some traditional machine learning methods is below 80%, while the LSTM, Gray+CNN, vectorCNN, and Cakir’s method all achieved over 90%. To better visualize the accuracy distribution of these models, the accuracy box-plots of these specific models are shown separately in Figure 10.

In addition, our work compares the models in terms of accuracy, precision, recall, and F1-score, taking the average of the results from ten experiments for each model. The comparison results are shown in Table 4. From the data, it can be seen that the proposed DEMI model achieved an accuracy of 97.30%, which is 0.83% higher than the Gray+CNN method, 0.73% higher than vectorCNN, 0.45% higher than Cakir’s method, and 0.08% higher than our previous work, MCIopcode2vec.

**Figure 11:**
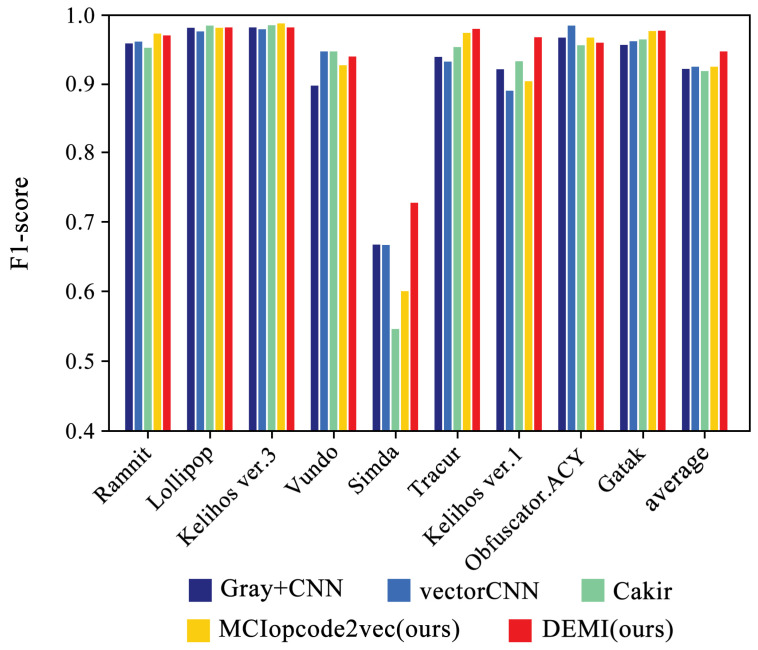
Family-wise F1-scores: DEMI vs. SOTA methods.

**Figure 12:**
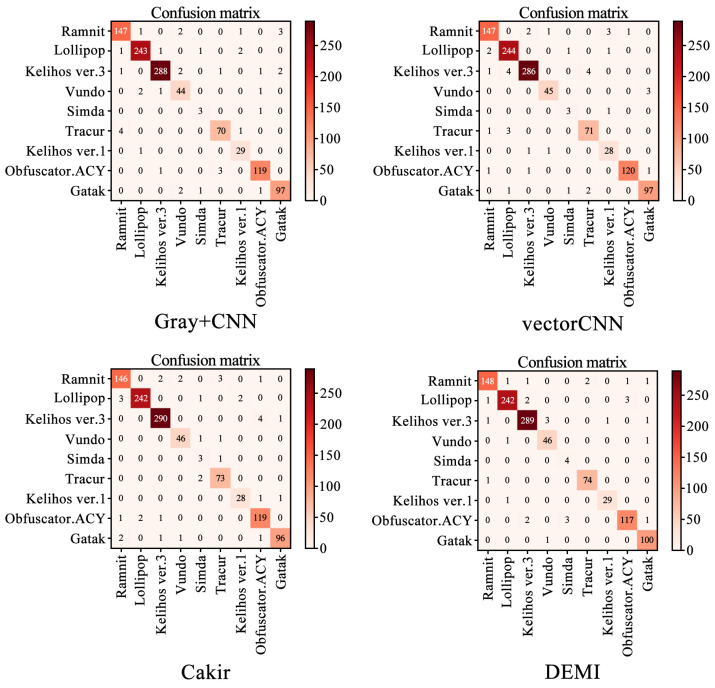
Confusion matrices for Gray+CNN, vectorCNN, Cakir’s method, and DEMI.

We selected four SOTA methods listed in Table 4 to compare with the proposed method. The identification accuracy for each malware family is shown in Figure 11. Additionally, the confusion matrices for Gray+CNN, vectorCNN, Cakir’s method, and DEMI are illustrated in Figure 12. It can be observed that DEMI significantly outperforms the other methods in terms of F1-score for the Simda family. For other families, the F1-scores are relatively close among all methods. Overall, DEMI achieves the highest average F1-score.

**Figure 13:**
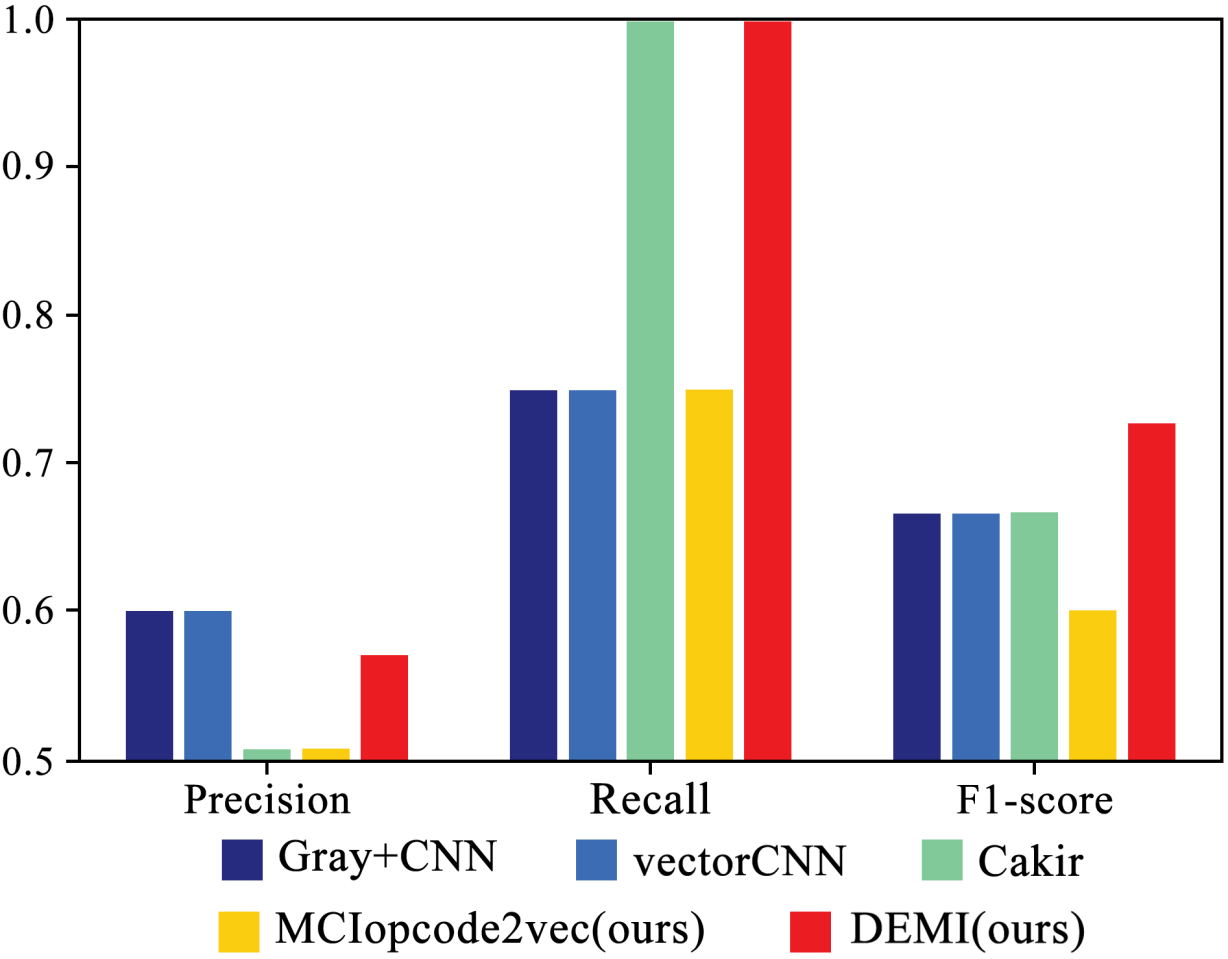
Performance comparison of DEMI and SOTA methods on the Simda family.

Figure 13 illustrates the detailed performance of four SOTA methods and the proposed DEMI on the Simda family. As shown, DEMI demonstrates superior precision, recall, and F1-score. These results suggest that DEMI, by generating adversarial samples for the Simda family, has effectively enhanced the dataset, leading to improved identification performance for Simda. Consequently, this enhancement results in a slightly better overall average performance compared with other methods.

#### 4.3.2. Comparison of Results for Few-Sample Training

**Figure 14:**
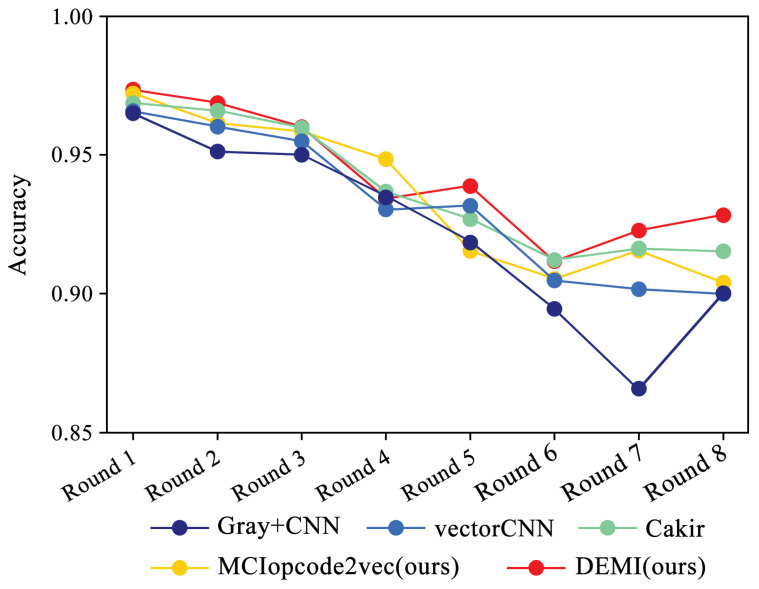
Relationship between sample number and accuracy.

In accordance with the rules described in the previous subsection, we progressively reduced the training samples to train and compare different models, maintaining the sample numbers for each round as consistent with Table 3. Figure 14 illustrates the overall identification accuracy trend for SOTA methods and the proposed DEMI across eight rounds of experiments. As the number of samples decreases, the identification accuracy for all models shows a downward trend. The comparison methods, including Gray+CNN, vectorCNN, Cakir’s method, and MCIopcode2vec, exhibit a significant decline in accuracy as the sample size reduces. Among these, DEMI demonstrates relatively more stable performance, maintaining high accuracy throughout the eight rounds. A noticeable drop in accuracy is observed in the sixth round, which we attribute to insufficient training of adversarial samples due to the reduced sample sizes in the Vundo and Kelihos ver.1 families, thus affecting the classifier’s accuracy.

**Figure 15:**
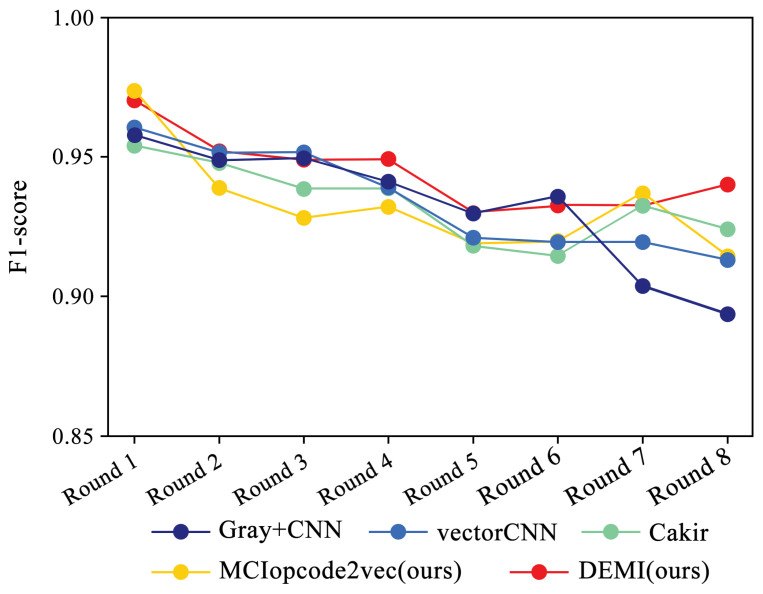
F1-score trends across eight rounds of few-sample experiments on Ramnit.

**Figure 16:**
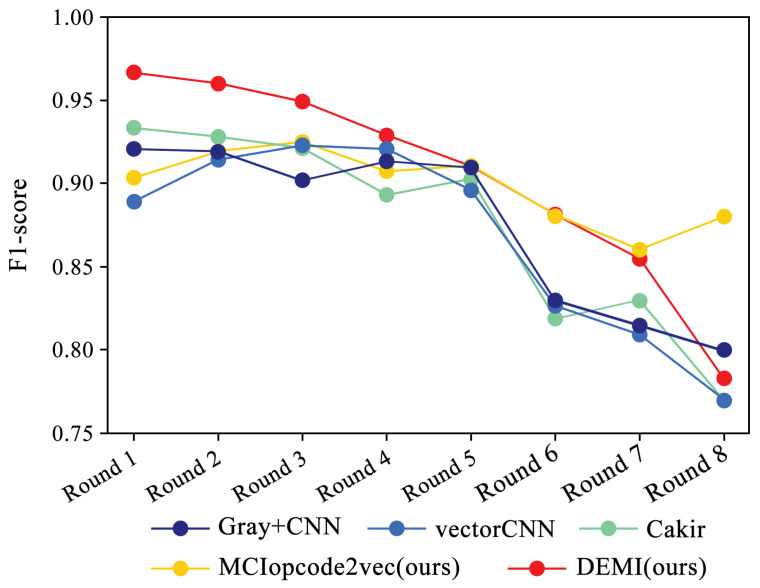
F1-score trends across eight rounds of few-sample experiments on Kelihos ver.1.

**Figure 17:**
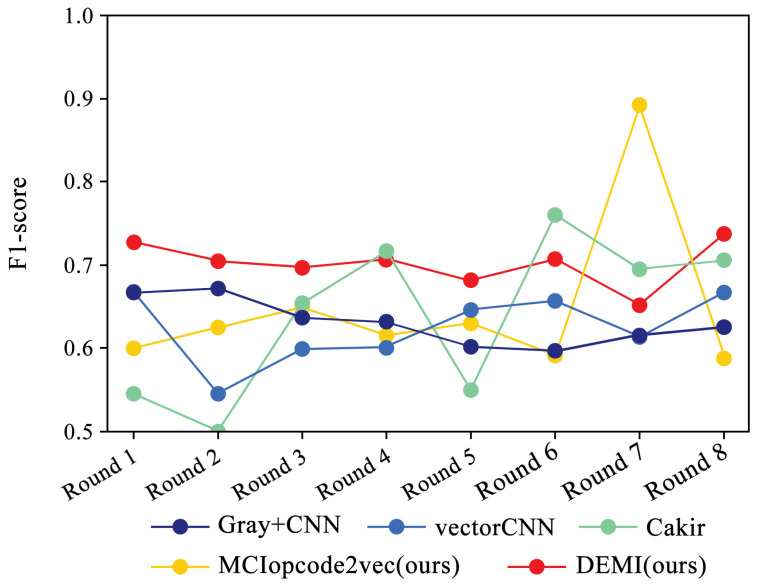
F1-score trends across eight rounds of few-sample experiments on Simda.

Additionally, we collected and analyzed the F1-score performance data for the Ramnit family with a large number of samples, the Kelihos ver.1 family with fewer samples, and the Simda family with special operations. The results are shown in Figure 15, Figure 16 and Figure 17, respectively.

Due to the ample number of samples in the Ramnit family, the DEMI model remains stable with a slight decline, whereas other models show a noticeable drop in F1-score performance. For the Kelihos ver.1 family, the DEMI model shows a general decline, with a sharp drop in the final round. We believe this is due to the small number of Kelihos ver.1 samples, leading to insufficient training of the adversarial network. Other models show a gradual decline in the early stages but a rapid decrease starting from the sixth round. Since the Simda family has a small total number of samples and a low recognition accuracy, we did not reduce the number of samples for this family to avoid affecting the overall accuracy. Even so, the final number of samples available for testing is only eight, making it highly susceptible to the reduction in samples in other families. Therefore, the Simda family exhibits fluctuations during training without a clear downward trend.

## 5. Discussion

Despite DEMI’s outstanding overall performance in both full-sample and few-sample scenarios, consistently leading across all experimental metrics, several challenges warrant further investigation. First, experiments on the Simda family indicate that when the original sample size is extremely small, both DEMI and other methods exhibit poor performance, highlighting the need for further optimization of the classifier model. Second, the current SOTA machine learning methods for ICSs lack consistent evaluation metrics, leading to discrepancies in performance results. Most experiments rely solely on accuracy, precision, recall, and F1-score for performance evaluation, with insufficient consideration of time-based metrics such as training and inference time, which are crucial for real-time identification applicability in ICSs. Finally, it is essential to validate and refine our method using more diverse datasets and through deployment in real-world ICS scenarios.

## 6. Conclusions

Our work presents a novel method for malware identification in ICSs, aiming to enhance the performance of malware classifiers. Existing identification methods often lack optimization for ICSs. Firstly, most current methods have insufficient training samples specific to ICSs, resulting in poor model accuracy. Secondly, the lack of diversity in training samples leads to classifiers’ instability and lack of robustness. We propose DEMI, which combines opcode2vec and CVAE-GAN to generate a large-scale and diverse malware sample set tailored for ICS. These enriched samples not only improve the performance of malware classifiers in detecting various types of malware in ICSs but also enhance their stability and robustness against metamorphic attacks. Consequently, the classifiers become more flexible and efficient in responding to threats.

## Figures and Tables

**Table 1:** Architecture of classifier based on DEMI.

Layer	Type	Kernel	Stride	Output Dimension
INPUT	Input Layer	-	-	266×266
CONV1	Convolutional Layer	5×5	2	32 filters
POOL1	Max Pooling Layer	2×2	2	-
CONV2	Convolutional Layer	5×5	2	32 filters
POOL2	Max Pooling Layer	2×2	2	-
CONV3	Convolutional Layer	5×5	2	64 filters
POOL3	Max Pooling Layer	2×2	2	-
FC1	Fully Connected Layer	-	-	128 neurons
FC2	Fully Connected Layer	-	-	9 neurons

**Table 2:** The number of samples of each family in each round of full-sample experiment.

Malware Family	Round 1		Round 2		Round 3		Round 4		Round 5	
	Real	Generated	Real	Generated	Real	Generated	Real	Generated	Real	Generated
Ramnit	1541	308	1541	370	1541	444	1541	533	1541	640
Lollipop	2478	496	2478	595	2478	714	2478	857	2478	1028
Kelihos ver.3	2947	589	2947	707	2947	848	2947	1018	2947	1222
Vundo	475	95	475	114	475	137	475	164	475	197
Simda	42	8	42	10	42	12	42	14	42	17
Tracur	751	150	751	180	751	216	751	259	751	311
Kelihos ver.1	298	60	298	72	298	86	298	103	298	124
Obfuscator.ACY	1228	246	1228	295	1228	354	1228	425	1228	510
Gatak	1013	203	1013	244	1013	293	1013	352	1013	422

**Table 3:** The number of samples of each family in each round of few-sample experiment.

Malware Family	Round 1	Round 2	Round 3	Round 4	Round 5	Round 6	Round 7	Round 8
Ramnit	1541	1233	986	789	631	505	404	323
Lollipop	2478	1982	1586	1269	1015	812	650	520
Kelihos ver.3	2947	2358	1886	1509	1207	966	773	618
Vundo	475	380	304	243	195	156	125	100
Simda	42	42	42	42	42	42	42	42
Tracur	751	601	481	385	308	246	197	157
Kelihos ver.1	298	238	191	153	122	98	78	62
Obfuscator.ACY	1228	982	786	629	503	402	322	258
Gatak	1013	810	648	519	414	331	266	212
Total	10,773	8626	6909	5536	4436	3557	2588	2292

**Table 4:** Comparison of different methods.

Method	Accuracy	Precision	Recall	F1-Score
*Traditional methods*				
RNN	0.9411	0.9001	0.9420	0.9206
GRU	0.9287	0.8952	0.9241	0.9094
LSTM	0.9330	0.8973	0.9352	0.9159
BiLSTM	0.9359	0.9075	0.9510	0.9287
Random Forest	0.9456	0.9011	0.9451	0.9226
*SOTA methods*				
Gray+CNN	0.9647	0.9064	0.9339	0.9199
vectorCNN	0.9657	0.9113	0.9346	0.9228
Cakir’s Method	0.9685	0.9092	0.9655	0.9365
MCIopcode2vec (ours)	0.9722	0.9061	0.9395	0.9225
*Proposed method*				
DEMI (ours)	**0.9730**	**0.9234**	**0.9744**	**0.9482**

**Bold** numbers indicate the best results.

## Data Availability

The original contributions presented in the study are included in the article; further inquiries can be directed to the corresponding author.
